# *Plasmodium falciparum* and *Mycoplasma pneumoniae* co-infection presenting with cerebral malaria manifesting orofacial dyskinesia and haemophagocytic lymphohistiocytosis

**DOI:** 10.1186/s12936-016-1517-x

**Published:** 2016-09-08

**Authors:** Praveen Weeratunga, Gowri Rathnayake, Ahalya Sivashangar, Panduka Karunanayake, Ariaranee Gnanathasan, Thashi Chang

**Affiliations:** 1 University Medical Unit, National Hospital of Sri Lanka, Colombo, Sri Lanka; 2 Department of Clinical Medicine, Faculty of Medicine, University of Colombo, 25, Kynsey Road, Colombo 08, Sri Lanka

**Keywords:** Malaria, Mycoplasma, Sri Lanka, Orofacial dyskinesia, Haemophagocytic lymphohistiocytosis

## Abstract

**Background:**

Malaria is a mosquito-borne infectious disease with diverse clinical manifestations caused by a parasitic protozoan of the genus *Plasmodium*. Complex inter-relationships between *Mycoplasma* species and *Plasmodium* parasites have been previously noted in vitro. This is the first report of *Plasmodium falciparum* and *Mycoplasma pneumoniae* co-infection in a human host presenting with cerebral malaria manifesting orofacial dyskinesias and haemophagocytic lymphohistiocytosis.

**Case presentation:**

A 55-year-old Sri Lankan man with a recent visit to South Africa presented with an acute febrile illness, cough and worsening dyspnoea with alveolar-interstitial infiltrates on chest radiography. Serological evaluation confirmed a diagnosis of *Mycoplasma* infection. He subsequently developed encephalopathy with orofacial dyskinesia. A diagnosis of severe *P. falciparum* infection with significant parasitaemia was established. Peripheral blood cytopaenia occurred due to haemophagocytic lymphohistiocytosis in the bone marrow. Complete clinical and haematological recovery was achieved with intravenous artesunate.

**Conclusions:**

*Plasmodium falciparum* and *Mycoplasma pneumoniae* co-infection occurring in vivo manifests clinical features that are plausibly a result of the interaction between the two microorganisms. This is the first report of orofacial dyskinesia in either infection.

**Electronic supplementary material:**

The online version of this article (doi:10.1186/s12936-016-1517-x) contains supplementary material, which is available to authorized users.

## Background

Malaria is caused by protozoa of the genus *Plasmodium,* of which five species are known to cause human disease; it is transmitted by *Anopheles* spp. mosquitoes. The clinical manifestations range from uncomplicated infection to severe multisystem affliction with significant mortality. *Plasmodium falciparum* infections have a high predilection of multisystem manifestations. This case report highlights the unusual combination of *Mycoplasma* and *P. falciparum* co-infection presenting with cerebral malaria manifesting orofacial dyskinesias and haemophagocytic lymphohistiocytosis.

## Case presentation

A 55-year-old Sri Lankan man employed as a chef in Dubai, United Arab Emirates, presented to the National Hospital of Sri Lanka with fever for five days and associated headache, arthralgia, myalgia and severe prostration approximately 3 weeks after spending 2 months in South Africa as part of his employment. He had been previously well and had not been on chemoprophylaxis for malaria while in South Africa. He has had a worsening non-productive cough and shortness of breath 1 day prior to presentation. On examination, he was pale and had posterior cervical lymphadenopathy. His respiratory rate was 30 cycles/min and he had bilateral diffuse scattered crepitations and rhonchi in both lung bases. Rest of the systems examination was normal.

Initial investigations revealed a marginal neutrophil predominance with thrombocytopaenia (platelets—55,000/mm^3^) and the chest radiograph showed alveolar-interstitial shadowing. The patient was empirically treated with clarithromycin for 7 days, which resulted in the resolution of the chest symptoms and signs, but with poor resolution of fever. His serum Mycoplasma IgM ELISA (Sigma Aldrich) antibody titre was strongly positive at a dilution of 1/1260 with a repeat titre noted at 1/7560, four weeks after the initial presentation. Given the recent travel history, a blood film was examined for evidence of malaria, but was noted to be negative. Despite initial improvement, progressive thrombocytopenia and worsening anaemia were noted from day 7 of his illness. By day 10, his level of consciousness deteriorated demonstrating abnormal behaviour and worsening drowsiness. Frequent, stereotyped, intermittent orofacial dyskinetic movements, not attributable to any of the drugs administered, were noted (see Additional file 1). Magnetic resonance imaging of the brain was normal. A blood film done at this time demonstrated multiple ring forms of *P. falciparum* with a parasite load of 300,000/μl. Intravenous artesunate was commenced and the patient demonstrated remarkable clinical improvement with resolution of abnormal movements and improvement of level of consciousness within 24 h. The high-spiking fever which was persistent up to day 12 of illness resolved. However a further decline in the platelet count was noted up to day 14 and a fall in the haemoglobin to 7 g/dl. His fasting triglyceride level was 405 mg/dl, serum ferritin 25,000 ng/ml and lactate dehydrogenase 1600 U/l. His INR was 1.7; APTT 41s; and fibrinogen level 1.1 g/l. Ultrasound scan of the abdomen demonstrated hepatosplenomegaly. Bone marrow examination demonstrated evidence of resolving haemophagocytosis.

These haematological manifestations subsequently resolved within the next 72 h and the patient made a complete clinical recovery.

## Conclusions

This patient highlights a rare combination of a dual-infection manifesting as a biphasic illness with clinical characteristics inherent to each microorganism. The initial presentation was of an acute febrile illness with predominant respiratory involvement and thrombocytopaenia with chest radiography demonstrating interstitial lung inflammation associated with serological confirmation of *Mycoplasma* infection. The subsequent phase was of worsening peripheral blood cytopaenia and neurological manifestations despite resolution of respiratory features caused by severe *P. falciparum* infection.

Complex inter-relationships between *Mycoplasma* species and *Plasmodium* parasites have been previously noted in vitro and in animal models [1, 2]. The effects of other microbial agents provoking host stress response to activate latent pathogenic *Mycoplasma* have been investigated in animal studies [1]. Accelerated growth of contaminant *Mycoplasma* species in cultures of *P. falciparum* had resulted in subsequent reduction in *Plasmodium* parasitaemia [2]. This is the first report of *P. falciparum* malaria and *Mycoplasma* co-infection in a human host. It is plausible that this patient acquired latent infections of both organisms in South Africa and the eradication of *Mycoplasma* may have led to a surge of *P. falciparum* parasitaemia, which had been suppressed by *Mycoplasma*, predisposing the patient to develop cerebral malaria. The incubation period for presentation in this patient is unusually long for *P. falciparum* malaria. The authors hypothesize that this long incubation period may be due to the suppression of *P. falciparum* parasitaemia due to *Mycoplasma* coinfection. It is extremely unlikely that he could have acquired the infection in Sri Lanka as the last endemic case was reported in October 2012.

The cerebral manifestations of *P. falciparum* infection are diverse, ranging from encephalopathy, seizures, dysconjugate ocular movements to psychiatric manifestations and post-malaria neurological syndromes [3]. However orofacial dyskinesias as observed in this patient have not been previously documented in malaria. Similarly, although neurological manifestations such as choreoathetosis have been reported in *Mycoplasma* infection [4], orofacial dyskinesia has not been described. It is plausible that the complex interaction between the two organisms may have predisposed to this atypical neurological manifestation, which is clinically significant since orofacial dyskinesia has been increasingly recognized to characteristically occur in NMDAR-antibody encephalitis [5, 6] and this case description adds to its differential diagnosis of a comatose patient presenting with movement disorders, particularly in the orofacial region.

The progressive cytopaenia in this patient was attributed to haemophagocytic lymphohistiocytosis (HLH). Although severe malaria *per se* can cause cytopaenia, HLH was considered more likely in this patient because the cytopaenia developed during the resolution phase of malaria and was associated with hypofibrinogenaemia, hyperferritinaemia, hypertriglyceridaemia and a high index of haemophagocytosis in the bone marrow. HLH has been previously reported with both *Mycoplasma* and malaria [7, 8]. Although other cytokine markers were not tested due to unavailability, this patient fulfilled the standard diagnostic criteria for HLH.

This is the first report of human *P. falciparum* and *Mycoplasma pneumoniae* co-infection manifesting clinical features that are plausibly a result of the interaction between the two microorganisms. It is also the first report of orofacial dyskinesia in either disease.

## Additional file

10.1186/s12936-016-1517-x Video demonstrating orofacial dyskinesia in cerebral malaria.

## Abbreviations

ELISA: enzyme linked immunosorbent assay
HLH: haemophagocytic lymphohistiocytosis
IgM: immunoglobulin M
